# Fostering Holistic Collaboration: Health Promotion with Physical Therapy and Speech-Language Pathology Students

**DOI:** 10.1093/geroni/igaf122.3265

**Published:** 2025-12-31

**Authors:** Mary Milidonis, Myrita Wilhite, Matthew Mino

**Affiliations:** Cleveland State University, Cleveland, Ohio, United States; Cleveland State University, Cleveland, Ohio, United States; Cleveland State University, Cleveland, Ohio, United States

## Abstract

This qualitative study explores the impact of interprofessional education (IPE) on physical therapy (PT) and speech-language pathology (SLP) students in health promotion clinics for older adults. By engaging in collaborative clinical practice, students from both disciplines were given the opportunity to work together in a real-world setting, promoting holistic care and fostering empathy. Thirty (26 DPT, 4 SLP) graduate students (mean age 24 years, 38.7% male) volunteered to answer open ended questions about an IPE experience for Brain Health Promotion with older adults. Teams screened older adults for cognition and endurance. Older adults were instructed in brain health activities. Three researchers reviewed student surveys with thematic analysis. The study highlights several key findings. First, students reported an enhanced ability to adapt their communication styles to ensure better understanding by older adult clients. Additionally, improved communication and collaboration among team members were noted, emphasizing the importance of interprofessional skills in delivering comprehensive care. Students also expressed increased confidence in clinical settings, attributing this to the hands-on experience and exposure to diverse perspectives. Moreover, the study identified the development of empathy as a crucial component of the learning process. Students recognized the importance of empathetic interactions in enhancing patient outcomes. Overall, the research suggests that IPE in health promotion clinics provides valuable benefits in cultivating essential skills for PT and SLP students, particularly in the context of working with older adults. These findings underscore the importance of incorporating IPE opportunities into educational programs to better prepare future clinicians for collaborative, patient-centered care.

